# Discontinued Drugs for the Treatment of Cardiovascular Disease from 2016 to 2018

**DOI:** 10.3390/ijms20184513

**Published:** 2019-09-12

**Authors:** Tingting Li, Sida Jiang, Bingwei Ni, Qiuji Cui, Qinan Liu, Hongping Zhao

**Affiliations:** 1 School of Pharmacy, China Pharmaceutical University, Nanjing 210009, Jiangsu, China; dreamerltt@163.com (T.L.); jstar20780@163.com (S.J.); 2 School of Engineering, China Pharmaceutical University, Nanjing 210009, Jiangsu, China; nibingwei123@163.com; 3 School of Science, China Pharmaceutical University, Nanjing 210009, Jiangsu, China; jicq96@163.com (Q.C.);

**Keywords:** OPC-108459, ONO-4232, GSK-2798745, LIK-066, TAK-536TCH, bococizumab

## Abstract

Cardiovascular drug research and development (R&D) has been in active state and continuously attracts attention from the pharmaceutical industry. However, only one individual drug can eventually reach the market from about the 10,000 compounds tested. It would be useful to learn from these failures when developing better strategies for the future. Discontinued drugs were identified from a search performed by Thomson Reuters Integrity. Additional information was sought through PubMed, ClinicalTrials.gov, and pharmaceutical companies search. Twelve compounds discontinued for cardiovascular disease treatment after reaching Phase I–III clinical trials from 2016 to 2018 are detailed in this manuscript, and the reasons for these failures are reported. Of these, six candidates (MDCO-216, TRV027, ubenimex, sodium nitrite, losmapimod, and bococizumab) were dropped for lack of clinical efficacy, the other six for strategic or unspecified reasons. In total, three candidates were discontinued in Phase I trials, six in Phase II, and three in Phase III. It was reported that the success rate of drug R&D utilizing selection biomarkers is higher. Four candidate developments (OPC-108459, ONO-4232, GSK-2798745, and TAK-536TCH) were run without biomarkers, which could be used as surrogate endpoints in the 12 cardiovascular drugs discontinued from 2016 to 2018. This review will be useful for those involved in the field of drug discovery and development, and for those interested in the treatment of cardiovascular disease.

## 1. Introduction

As the number 1 cause of death globally, cardiovascular diseases (CVDs) take the lives of 17.7 million people every year, about 31% of all deaths worldwide. CVDs are disorders of the heart and blood vessels, which include coronary heart disease, cerebrovascular disease, rheumatic heart disease, and other conditions [1]. Cardiovascular drug research and development (R&D) has been in active state and continuously attracts attention from the pharmaceutical industry. However, only one individual drug that start preclinical or clinical development can eventually reach the market among the ~10,000 compounds tested.

Identifying a drug project that carries a high risk of imminent failure as early in the development as possible is critical for capital efficiency. Since the investment in clinical trials is just a portion of the full picture of drug development, the actual impact is usually considerably higher. Financial investment in failed projects is often factored in the cost of an average drug development. The cost of R&D of discontinued candidates reflects the price of the drugs that do reach the market. If a drug that is deemed to be discontinued, the earlier the discontinuation, the fewer the adverse events and the expenses for the process of drug development. Therefore, it would be helpful to recapitulate previous failures, report the reason for discontinuation, and give some meaningful advice.

In the past years, the discontinued drugs for cardiovascular disease treatment are overall declining. There were 30 candidates in 2012, 11 candidates in 2013, and four candidates in 2014. The failures were due to efficacy, safety, strategic factors, and unknown reasons. In addition, the major reasons were efficacy and strategic factors [2,3,4,5]. Strategic re-evaluation focus has been one the major reasons for different drug developments’ discontinuation over the years. While efficacy reasons are generally straightforward, reasons of termination due to strategy maybe complex [6]. Some discontinued drugs have been demonstrated to be effective and safe, but the changed internal and external environment of pharmaceutical companies and the competition in the market, or recruitment failure would lead pharmaceutical companies to terminate the development. Therefore, these drug candidates may possess development value in the future.

As the history of a failure can indicate a more promising way to a success, the aim of this review was to clarify the drug candidates for the treatment of cardiovascular disease discontinued after reaching clinical trials from 2016 to 2018, the phase of discontinuation, and the reason for it. There were 12 cardiovascular drugs discontinued in the last 3 years, according to the search results derived by setting milestone = “Discontinued”, milestone date = from 20160101 to 20181231, and therapeutic group = “CARDIOVASCULAR DRUGS”, and afterward filtering out some drugs that were not discontinued because of cardiovascular disease indications from Thomson Reuters Integrity, which stays at the cutting edge of drug R&D and provides information integrated from multiple fields of drug R&D, including every significant new drug under development from lead through early preclinical study and clinical phases, to launched or discontinued status and beyond [7]. Additional information was sought through PubMed, ClinicalTrials.gov, and pharmaceutical companies’ search.

## 2. Discontinued Drugs

### 2.1. General Overview

According to Table 1, 12 drug candidates for the treatment of cardiovascular disease were removed from the CVD development pipeline from 2016 to 2018. Of these, three candidates were dropped in Phase I trials, six in Phase II, and three in Phase III. The detailed information of the 12 drugs is given below.

### 2.2. Discontinued Drugs in Phase I

#### 2.2.1. PF-06282999

The thiouracil derivative PF-06282999 is a myeloperoxidase (MPO) inhibitor [8]. As an important member of the haem peroxidase-cyclooxygenase superfamily, MPO is a key factor in a great number of conditions within the group of cardiovascular diseases, neurodegenerative diseases, immune-mediated diseases, etc. [9]. Preclinical studies showed that PF-06282999 was rapidly and well absorbed in mice, rats, dogs, and monkeys with oral bioavailability values of 100%, 86%, 75%, and 76%, respectively. It was well distributed with steady state distribution volumes ranging from 0.5–2.1L/kg in the four species following intravenous administration (iv) [10]. PF-06282999 showed moderate permeability via both paracellular and transcellular pathways, and is a substrate for multidrug resistance protein 1 (MDR1) based on the bidirection transport study with a low efflux ration of 3.3. Renal clearance of unchanged PF-06282999 emerged as the principal clearance mechanism in humans other than hepatic clearance involving metabolizing enzymes and drug transporters [8]. Although PF-06282999 exhibited no relevant reversible and time- or NADPH-dependent inhibitory effects against human CYP450 enzymes in vitro, studies using human hepatocytes revealed moderate induction of CYP3A4 mRNA levels and midazolam-1′-hydroxylase activity in a PF-06282999 dose-dependent fashion, which is mediated by the pregnane X receptor [11,12]. The Phase I trial study showed that the magnitude of the 4β-hydroxycholesterol or 6β-hydroxycortisol ratio change (endogenous biomarkers of CYP3A4) was generally smaller than the magnitude of an oral midazolam area under the curve (AUC) change with PF-06282999, so an oral midazolam drug–drug interaction (DDI) study is ultimately needed to gauge DDI via CYP3A4 induction, which is likely to be the case for weak to moderate CYP3A4 inducers or inducers that have a significant intestinal CYP3A4 induction component [13]. PF-06282999 was in Phase I clinical trials at Pfizer for the treatment of acute coronary syndrome [14]. However, product development for this indication was discontinued in the second quarter of 2017 with no reason.

#### 2.2.2. OPC-108459

Otsuka Pharmaceutical Co., Ltd. was previously developing OPC-108459 for the treatment of paroxysmal and persistent atrial fibrillation. A Phase I trial study, which enrolled 48 patients with paroxysmal or persistent atrial fibrillation, was carried out to investigate the safety, pharmacokinetics (PK), and efficacy of OPC-108459 following 30-min continuous iv administration at 0.4, 0.8, 1.6, 2.4 mg/kg, or placebo [15]. However, the development of the product was discontinued due to insufficient scientific evidence obtained in the Phase I clinical trials in 2016.

#### 2.2.3. ONO-4232

As a prostanoid EP4 agonist, ONO-4232 improves left ventricular diastolic dysfunction and ameliorates acute and chronic heart failure in animal models [16]. It had been in Phase I clinical trials at Ono Pharmaceutical Co., Ltd. for the treatment of acute heart failure (AHF). The data showed that the plasma concentrations of ONO-4232 reached steady state approximately 2 h after the start of infusion and then declined rapidly after the end of infusion, and systemic exposure appeared to increase in a dose-proportional manner. Approximately 30% of the administered dose of ONO-4232 was excreted in the urine. ONO-4232 is generally well tolerated in the dose range of 0.001 to 0.27 ng/kg/min. This Phase I clinical study supported further evaluation of the cardiovascular effects of this first-in-class selective left ventricular lusitropic and venodilatory drug in patients with acutely decompensated heart failure [17]. However, Ono discontinued the development of ONO-4232 due to a strategic reason in January 2016 [18].

### 2.3. Discontinued Drugs in Phase II

#### 2.3.1. GSK-2798745

As a transient receptor potential cation channel subfamily V member 4 (TRPV4) antagonist, GSK2798745 was developed as a novel therapeutic intervention for the treatment of pulmonary edema associated with heart failure (HF) [19]. It may improve pulmonary function, respiration, and gas exchange, as well as sleep-disordered breathing in patients with heart failure.

A Phase I trial was conducted for the PK of three 2.4 mg tablet formulations of GSK2798745, which had been completed on December 2016 [20]. Single and repeat oral doses of GSK2798745 were given to healthy subjects and stable HF subjects to study the safety, tolerability, PK, and pharmacodynamics (PD) in a Phase II trial study [21]. A randomized, double-blind, sponsor-unblinded, placebo-controlled, Phase 2a crossover study in adults with heart failure had been completed by August 21, 2017, which was designed to investigate the effect of repeat administration of GSK2798745 on pulmonary gas exchange and pulmonary function [22]. However, GlaxoSmithKline (GSK) terminated the development of this product for unspecified reasons [23].

#### 2.3.2. MDCO-216

MDCO-216, a recombinant apolipoprotein A-I (ApoA-I) Milano (AIM) and phospholipid complex, increases high-density lipoprotein (HDL)-cholesterol by mimicking HDL and its function, removing cholesterol and other lipids from tissues including the arterial wall and transporting them to the liver for elimination in reverse lipid transport [24].

In a preclinical study, MDCO-216 was administered to cynomolgus monkeys at 30, 100, and 300 mg/kg every other day for a total of 21 infusions, and effects on lipids, (apo) lipoproteins, and ex-vivo cholesterol efflux capacity were monitored. The change in lipid and lipoprotein profile sustained over a six-week period was well tolerated and did not lead to any obvious adverse effects [25]. Twenty-four healthy volunteers and 24 patients with documented coronary artery disease (CAD) received a 2 h infusion of MDCO-216 in a randomized, placebo controlled, single ascending dose from 5–40 mg Phase I trial study. The studies showed that MDCO-216 was well tolerated with no significant safety findings both in healthy and CAD patients and modified key lipid-related biomarkers [26,27,28]. A double-blind, placebo controlled Phase II clinical trial, which enrolled 126 patients with acute coronary syndrome (ACS) was carried out to measure the effect on atherosclerotic plaque burden and the impact on cholesterol efflux by intravascular ultrasound (IVUS). The patients received weekly 20 mg/kg MDCO-216 infusions over five weeks period [29]. Phase I and II trials from December 2015 to October 2016 showed that the agent did not ameliorate intracoronary atherosclerotic plaque enough for its further development, which led to a termination of its further study for atherosclerosis in November 2016 [30].

#### 2.3.3. TRV027

As a first-in-class biased G protein-coupled receptor (GPCR) ligand that targets β-arrestin-biased angiotensin II type 1 receptor, TRV027 is a novel agent with vasodilating properties, which may improve both in-hospital and post-discharge clinical outcomes for patients with AHF [31].

In preclinical studies, TRV027 showed a dose-dependent decrease in mean arterial pressure and pulmonary capillary wedge pressure while preserving renal function (even in animals that were administered with furosemide). Apparent blood pressure (BP) effects of TRV027 were rapid in onset and relatively short in duration, providing an ideal hemodynamic profile for use in AHF [32,33,34]. A Phase I trial demonstrated that TRV027 was safe and well tolerated with a short-half-life (ranging between 2.4 and 13.2 min) and dose-proportional increased in systemic exposure. Meanwhile, TRV027 could reduce BP to a greater degree in subjects with renin angiotensin system (RAS) activation than in those with normal plasma renin activity (PRA) levels [35]. Early clinical studies had been conducted by Trevena Inc. for the treatment of acute decompensated heart failure [36,37]. In May 2013, a license option agreement was signed by Trevena Inc. to Forest Laboratories for exclusive worldwide development following the completion of Phase IIb [38]. Biased Ligand of the Angiotensin Receptor Study in AHF (BLAST-AHF) was designed to determine the safety, efficacy, and optimal dose of TRV027 to advance into future studies. A multi-center, international, randomized, double-blind, placebo-controlled, parallel group, Phase IIb dose-ranging study, enrolled 621 patients with AHF. TRV027 did not improve clinical status through 30-day follow-up compared with placebo [39,40]. In 2017, discontinuation of TRV027 was scheduled by Trevena for the treatment of acute decompensated HF.

#### 2.3.4. Ubenimex

Ubenimex is an oral, small-molecule dual-inhibitor of aminopeptidase and leukotriene hydrolase, immunostimulant isolated from the fermentation broth of Streptomyces olivoreticuli by the Institute of Microbial Chemistry [41]. Nippon Kayaku Co., Ltd. firstly developed ubenimex as an adjunct to chemotherapy to extend survival and to maintain remission after treatment for acute non-lymphocytic leukemia in adults. Ubenimex was developed by Eiger BioPharmaceuticals for pulmonary arterial hypertension (PAH) as well as other inflammatory diseases involving leukotriene B4 (LTB4).

A preclinical study showed that ubenimex (bestatin), injected into the carotid artery, inhibited the activity of aminopeptidase and enkephalinase, producing an accumulation of enkephalins in the central nervous system. Enkephalins activate opioidergic receptors in the brain, but concomitantly produce a depression of the cholinergic–adrenergic interaction in the central nervous system, which is known to be a prerequisite for the hypertensive response to physostigmine in the rat [42]. In the SU5416/athymic rat model of severe pulmonary hypertension (PH), bestatin was highly effective in reversing PH in dose-dependent. Meanwhile, bestatin prevented PH-related death, opened the obstructed arterioles, and improved cardiac function. An inhalable formulation of bestatin also reversed established PH [43]. A randomized, double-blind, placebo-controlled Phase II trial (EIG-UBX-001, LIBERTY) in 61 pulmonary arterial hypertension patients in Canada and the US, to evaluate the efficacy, safety, and tolerability of ubenimex 150 mg capsule three times a day, administered orally for 24 weeks, was completed [44]. Bestatin could inhibit HPAAF proliferation, migration, and differentiation by down-regulating p38 MAPK as well as Nox4 signaling pathways. In an autoimmune model of PH, inhibition of these pathways blocked perivascular inflammation, decreased Nox4 expression, reduced reactive oxygen species production, reversed arteriolar adventitial fibroblast activation, and attenuated PH development [45]. However, an open-label, single group Phase II trial (EIG-UBX-002, Liberty2) in Canada and the US, in 51 patients with PAH, to evaluate the long-term safety and efficacy of ubenimex po 150 mg, was terminated following the failure to demonstrate efficacy in EIG-UBX-001 [46].

#### 2.3.5. LIK-066

LIK-066, an oral sodium-dependent glucose cotransporter (SGLT)-1 and -2 inhibitor, has been developed by Novartis for the treatment of obesity, non-alcoholic steatohepatitis, and type 2 diabetes. A multi-center, double-blind, parallel-group dose-finding Phase II study was carried out in 125 type 2 diabetes mellitus patients with HF to assess the efficacy, safety and tolerability of three doses of LIK066 compared to placebo or empagliflozin. However, the study was prematurely discontinued on May 4, 2018 due to slow enrollment that would preclude obtaining study results in a timely manner [47].

#### 2.3.6. Sodium Nitrite

Savara Inc. developed a sodium nitrite inhalation solution as Aironite or AIR for treatment PAH and HF with preserved ejection fraction (HFpEF). In preclinical experiments of mice and rats with hypoxia or monocrotaline-induced PAH, low doses of nebulized nitrite (1.5 mg/min) one or three times a week could minimally increase plasma and lung nitrite levels and prevent or reverse PAH and pathological right ventricular hypertrophy and failure. In vitro and in vivo studies revealed that xanthine oxidoreductase was responsible for hypoxic metabolism of nitric oxide in the lung. Furthermore, physiological levels of nitrite inhibited hypoxia-induced proliferation of cultured pulmonary artery smooth muscle cells via the nitric oxide-dependent induction of the cyclin-dependent kinase inhibitor p21Waf1/Cip1 [48]. Inhaled nitrite of newborn lambs with PH induced by hypoxia elicited a rapid and sustained reduction (approximately 65%) in hypoxia-induced PH, which was deoxyhemoglobin- and pH-dependent, and associated with increased blood levels of iron-nitrosyl-hemoglobin [49].

The Phase I studies of inhaled nebulized sodium nitrite were carried out in a total of 82 healthy male and female subjects under normal and mildly hypoxic conditions, and following co-administration with steady-state sildenafil. Nebulized sodium nitrite was well tolerated following 6 days of every 8 h administration up to 90 mg, producing significant increases in circulating iron-nitrosyl hemoglobin, S-nitrosothiols, and the fraction exhaled nitric oxide. Pulmonary absorption of nitrite was rapid and complete, and the plasma exposure dose was proportional through the maximum tolerated dosage level of 90 mg, without accumulation following repeated inhalation. At higher dosage levels, dose-limiting toxicity were orthostasis (observed at 120 mg) and hypotension with tachycardia (at 176 mg), but venous methemoglobin did not exceed 3.0% at any time in any subject. Neither the tolerability nor PK of nitrite were impacted by conditions of mild hypoxia, or co-administration with sildenafil, supporting the safe use of inhaled nitrite in the clinical setting of PAH [50,51,52]. A single-center, pilot trial of low dose sodium nitrite (1 or 9.6 mg dose) vs. placebo Phase I study was carried on the 11 hospitalized out-of-hospital cardiac arrest (OHCA) patients. Compared to placebo, infusion of low doses of sodium nitrite in comatose survivors of OHCA had no significant effects on heart rate within 30 min after infusion, systolic blood pressure, or methemoglobin levels. A 9.6 mg sodium nitrite dose could produce significant C_15min_ plasma nitrite elevations to 2–4 μM [53,54].

A preliminary Phase II study of AIR001 for the treatment of PAH with 29 WHO Group 2 patients was finished to evaluate whether AIR-001 may be effective in reducing pulmonary vascular resistance (PVR), pulmonary capillary wedge pressure (PCWP), and right atrial pressure (RAP). The data demonstrated improvements in hemodynamic parameters and exercise capacity, and AIR001 was well tolerated [55]. An open-label, randomized, parallel-dose Phase II trial (AIR001-CS05) will be conducted to determine the safety and efficacy of nebulized sodium nitrate 80 mg once-daily, 46 mg or 80 mg four times daily over 16 weeks in 29 subjects with WHO group 1 PAH by monitoring changes in PVR—from baseline/day 1 (start of study drug) to the end of the study. However, it has been terminated due to acquisition of sponsor and change in corporate priorities [56]. Another Phase II trial (AIR001-CS06) to evaluate the intermediate/long-term safety and efficacy of nebulized AIR-001 who have completed the 16 week AIR001-CS05 trial in 17 patients has been terminated due to the same reason [57]. In a double-blind, randomized, placebo-controlled, parallel-group Phase II trial, 26 subjects with HFpEF underwent cardiac catheterization with simultaneous expired gas analysis at rest and during exercise, prior to and following treatment with inhaled sodium nitrite (90 mg) or placebo. Inhaled nitrite reduced resting PCWP, improved pulmonary artery compliance and decreased mean pulmonary artery pressures at rest and with exercise. Nitrite reduced RAP with no effect on cardiac output or stroke volume. Acute administration of inhaled sodium nitrite reduces biventricular filling pressures and pulmonary artery pressures at rest and during exercise in HFpEF [58,59]. To determine the effect of chronic administration of inhaled sodium nitrite on exercise capacity in HFpEF, a placebo-controlled, 2-treatment, crossover trial of 105 patients with HFpEF was designed. There were no significant between-treatment phase differences in daily activity levels, Kansas City Cardiomyopathy Questionnaire Clinical Summary Score, functional class, echocardiographic E/e’ ratio, or N-terminal fragment of the prohormone brain natriuretic peptide levels. These results showed that administration of inhaled inorganic nitrite for 4 weeks did not result in significant improvement in exercise capacity [60,61]. Savara Inc. discontinued the development of sodium nitrite for the negative outcome from inorganic nitrite delivery to Improve Exercise Capacity (INDIE) study in HFpEF [62].

### 2.4. Discontinued Drugs in Phase III

#### 2.4.1. TAK-536TCH

TAK-536TCH is a fixed-dose combination of azilsartan (AZI), amlodipine (AML) and hydrochlorothiazide (HCTZ) developed by Takeda Pharmaceutical Co., Ltd. for the treatment of essential hypertension [63]. A randomized, crossover Phase I trial study to evaluate the food-effect on the PK and safety of a single oral dose of TAK-536TCH tablet under fasted and fed conditions in the morning in 12 healthy adult male subjects was completed in 2016 [64]. Finished in 2013, a Phase II/III trial study, which enrolled 353 patients with grade I or II essential hypertension by 10-week treatment of TAK-536TCH, showed that TAK-536TCH (AZI/AML/HCTZ 20/5/12.5 mg) led to a significantly greater reduction in BP than the dual therapy (TAK-536CCB, AZI/AML), and was well tolerated in Japanese patients [65,66]. Since 2014, Phase III, open-label, multicenter clinical study was carried out on 341 patients with essential hypertension, which comprised a 4-week run-in period and 52-week treatment period. The primary and secondary endpoints were long-term safety and BP (office and home), respectively. Triple combination therapy with a single tablet of 20 mg AZL, 5 mg AML, and 12.5 mg HCTZ was well tolerated, and provided consistent BP-lowering effects for patients with essential hypertension whose BP was not adequately controlled by dual combination therapy with 20 mg AZL and 5 mg AML [67,68]. However, its preregistration for essential hypertension was canceled due to insufficient new value provided in the Japanese market in May 2017 [63].

#### 2.4.2. Losmapimod

Developed by GSK, losmapimod is an inhibitor of p38 mitogen-activated protein kinases (MAPKs) α and β isoforms. P38 MAPKs determine the transcription and translation of inflammatory mediators like tumor necrosis factor alpha (TNF-α) and interleukins 1 (IL-1), which are activated in atherosclerotic disease [69]. Furthermore, they undermine NO bioavailability by influencing the formation of reactive oxygen species resulting in vasoconstriction and endothelial dysfunction.

In preclinical studies, losmapimod could significantly and dose-dependently improve survival and endothelial function, and attenuated hypertension, cardiac remodeling and interleukin-1β (IL-1β) in the spontaneously hypertensive stroke-prone rat (SHR-SP) [70]. Losmapimod progressively suppressed dynamin-like protein 1(DLP1)/mitochondrial fission factor (MFF) from 6 h to 24 h after ischemia-reperfusion injury of rats, which suggested potential neuroprotective effect via suppressing mitochondrial fragmentation/mitophagy in stroke [71].

A single-center, single blind, Phase I and two-part study characterized the safety, tolerability, PK and PD of losmapimod and its metabolite GSK198602 in healthy Japanese volunteers. Losmapimod was found safe and well-tolerated following single doses (2.5 mg, 7.5 mg, 20 mg) and repeat doses (7.5 mg for 14 days) of oral administration in healthy Japanese volunteers, and no serious adverse events occurred during the study. The Tmax of losmapimod was 3–4 h, and the mean terminal elimination half-lives (T1/2) was approximately 7.9–9.0 h [72,73]. Another Phase I clinical study completed in 2010 showed that single iv infusion of losmapimod in healthy volunteers was safe and well tolerated, and may potentially serve as an initial loading dose in ACS as rapid exposure. A direct-link maximal inhibitory effect model related plasma concentrations to pHSP27 concentrations [74,75]. The population PK/PD meta-analysis of Phase I clinical studies in healthy volunteers indicated that losmapimod plasma concentration had no significant effect on QT-interval prolongation [76]. A total of 535 non-ST-segment elevation myocardial infarction (NSTEMI) patients were enrolled to receive oral losmapimod or matching placebo for 12 weeks in Phase II trial study (SOLSTICE trial, study of losmapimod treatment on inflammation and infarct size), which was confirmed the safety and efficacy of losmapimod in NSTEMI patients. Losmapimod was well tolerated in NSTEMI patients according to adverse events, alanine aminotransferase (ALT) concentrations, and cardiac events, and might improve outcomes after ASCs (MRI recruitment target, infarct size, left ventricular ejection fraction, left ventricular remodeling). Meanwhile, high-sensitivity C-reactive protein (hsCRP), B-type natriuretic peptide (BNP), and IL-6 were lower in losmapimod arma than placebo [77,78]. Based on the promising preliminary data of the Phase II SOLSTICE study, 3503 patients hospitalized with NSTEMI or STEMI were further enrolled in the Phase III (LATITUDE-TIMI 60) study to evaluate its effects in patients with ACS, which was completed in December, 2015. The primary endpoint (cardiovascular death, MI or severe recurrent ischemia requiring urgent coronary artery revascularization) occurred by 12 weeks in 123 patients treated with placebo (7.0%) and 139 patients treated with losmapimod (8.1%). The on-treatment rates of serious adverse events were 16.0% with losmapimod and 14.2% with placebo [79,80,81]. According to the unmet primary endpoint of its efficiency in Phase A of the trial and inadequate support to investment in the part B of the study, the further investigation of losmapimod for ACS was discontinued in January 2016 [82].

#### 2.4.3. Bococizumab

As a proprotein convertase subtilisin–kexin type 9 (PCSK9) inhibitor, bococizumab lowers low-density lipoprotein (LDL) level. PCSK9 is a serine protease, which bonding to LDL receptor (LDL-R) results in the aggregation of LDL cholesterol (LDL-C), an important factor causing atherosclerotic heart disease. Working as a humanized monoclonal antibody (mAb), bococizumab extracellularly influences circulating PCSK9 either by secluding it or blocking the bonding [83,84].

In Phase I studies, treatment with bococizumab by single or multiple IV or SC doses could lead to significant low-density lipoprotein cholesterol (LDL-C) reductions compared with placebo (43–84%) [85,86,87,88]. Bococizumab was generally safe and well tolerated in the short duration studies, both in diet-managed hypercholesterolemic subjects and in those on concomitant atorvastatin therapy, which was in line with results of the Phase II studies [89]. Peak plasma concentrations (Cmax) and T1/2 of bococizumab increased in a dose-dependent manner. The relative bioavailability for the 200 mg SC dosages ranged 22–26%. Multiple-dose study indicated that dose-related accumulation was minimal by weekly IV infusions of bococizumab, which geometric mean observed accumulation ratios (Rac) were 1.3, 1.7, 1.8, and 2.0 for the 0.25, 0.5, 1, and 1.5 mg/kg groups, respectively. A mechanism-based drug–target binding model using bococizumab Phase I and IIA trial data was developed to account for bococizumab, PCSK9, and LDL-C concentrations, and the effects of co-administration of statins. The model indicated that statins could predict an increase in LDL-C clearance of bococizumab by co-administration, which was consistent with the proposed effect of statins in stimulating both LDLR and PCSK9 production and the target-mediated disposition of bococizumab [83,90]. In July 2016, a Phase I trial for hypercholesterolemia in combination with recombinant human hyaluronidase (rHuPH20) was terminated due to business priorities regarding study execution [91,92].

The six SPIRE (studies of PCSK9 inhibition and the reduction of vascular events) lipid-lowering trials of Phase III enrolled 4300 patients with hyperlipidemia. Bococizumab showed wide variation in the LDL-C, which was present as early as 12 weeks (largely before the detection of antidrug antibodies) and also at 52 weeks among patients who did not have an antidrug-antibodies response [93,94,95]. Another two SPIRE trials of Phase III (multi center, double blind, randomized, placebo controlled, parallel group) study involved 27,438 patients with high cardiovascular risk to evaluate the efficacy, safety, and tolerability of bococizumab, in reducing the occurrence of major cardiovascular events. Compared to placebo, bococizumab had no benefit with respect to the primary end point of major adverse cardiovascular events in SPIRE-1 trial involving lower-risk patients. In contrast, a significant benefit in the SPIRE-2 trial involving higher-risk patients was shown [96,97]. Based in part on the high rate of immunogenicity of the drug, as well as on the wide variation in the LDL-C response among the patients, Pfizer discontinued global development of bococizumab because the totality of clinical information indicated almost no possibility for bococizumab to provide value to patients, physicians and shareholders.

## 3. Expert Opinion

These 12 drugs discontinued from 2016 to 2018 together with the 17 drugs discontinued in 2013–2015 were the cardiovascular drugs dropped from the global cardiovascular drug development pipeline in the past 6 years [4,5]. Each year less than five cardiovascular drugs were discontinued after reaching Phase I–III clinical trials, which was significantly fewer than the 30 discontinuations reported in 2012 [3] and 19 discontinuations in 2011 [2]. The trend of increasing numbers of cardiovascular drug development terminations in recent years has changed.

Of the 12 drugs failed from 2016 to 2018, the most eye-catching three drugs are ONO-4232, LIK-066, and bococizumab, which are both new chemical entities (NCE) without patent protection. Ono Pharmaceutical Co., Ltd. discontinued the developments of ONO-4232 for strategic reasons. LIK-066 was prematurely discontinued by Novartis due to slow enrollment, which would preclude obtaining study results in a timely manner. Developments of these three candidates still deserve further concerns. In addition to alirocumab and evolocumab for cholesterol-lowering and evolocumab for the prevention of heart attack and stroke, which are the only two approved inhibitors of PCSK9, bococizumab is another monoclonal antibody against PCSK9 [98,99]. As its mode of action differs from that of statins, anti-PCSK9 may provide benefit to people who do not achieve desirable LDL-C levels with statins or cannot tolerate them.

Among the failed cardiovascular drugs from 2016 to 2018, six drugs were dropped for lack of clinical efficacy, demonstrating the need for the development of more predictive animal models, and the need to develop experimental medicine paradigms that are more predictive of outcomes and to carry out such proof-of-concept clinical trials much earlier in development, especially during first-in-man studies [100]. With the development of artificial intelligence (AI) assistant drug designing, the problem for poor efficacy or safety may be solved by deep learning technology to predict and optimize the properties of the candidates.

It was reported that the success rate of drug R&D utilizing selection biomarkers is higher [101]. There are four candidate developments (OPC-108459, ONO-4232, GSK-2798745, TAK-536TCH) run without biomarkers, which could be used as surrogate endpoints in the 12 cardiovascular drugs discontinued from 2016 to 2018. In order to reduce the attrition rate of cardiovascular drug R&D, trials run with biomarker-selected patients should be given priority to study.

Overall, the range of the discontinued compounds discussed offers a relevant picture of what is currently in the pipeline for the CVD conditions such as those mentioned in this retrospective review. Future research may benefit from these developments and investigators conducting similar studies may learn from these failures.

## Figures and Tables

**Table 1:** Discontinued drugs for the treatment of cardiovascular disease from 2016 to 2018.

Drug Name(s), Structure	Organization	Mechanism of Action	Therapeutic Group	Development Phase Reached	Reason for Discontinuation	Discontinued Indications
PF-06282999 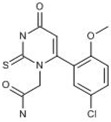	Pfizer (Originator)	Myeloperoxidase Inhibitors	Treatment of Disorders of the Coronary Arteries and Atherosclerosis	I	Unspecified	Acute coronary syndrome
OPC-108459 *	Otsuka Pharmaceutical (Originator)	Unknown	Antiarrhythmic Drugs	I	Miscellaneous	Atrial Fibrillation, atrial
ONO-4232 *	Ono (Originator)	Signal Transduction Modulators; Prostanoid EP4 Receptor Agonists	Heart Failure Therapy	I	Strategic	Heart failure
GSK-2798745; 2798745 *	GlaxoSmithKline (Originator)	TRPV4 Antagonists	Edema, Treatment of; Heart Failure Therapy	II	Unspecified	Heart failure, congestive
MDCO-216 *; AIM; ApoA-1 Milano; ESP-24217; ETC-216; Recombinant ApoA-I Milano/phospholipid complex	The Medicines Co.; Esperion Therapeutics, Pfizer (Originator)	HDL-Cholesterol Increasing Agents	Restenosis Treatment of; Atherosclerosis Therapy; Treatment of Disorders of the Coronary Arteries and Atherosclerosis; Lipoprotein Disorders, Treatment of; Cardiovascular Diseases (Not Specified)	II	Efficacy	Atherosclerosis
TRV027; TRV120027 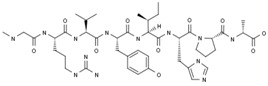	Trevena (Originator)	Signal Transduction Modulators; Angiotensin AT1 Receptor Ligands	Heart Failure Therapy	II	Efficacy	Heart failure, acute decompensated
Ubenimex; bestatin 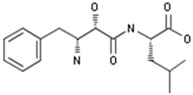	Nippon Kayaku	Immunostimulant; Peptidase inhibitor; Leucotriene B4 antagonist; Hit substrate-selective leucotriene A4 hydrolase inhibitor	Lung Cancer Therapy; Leukemia Therapy; Immunostimulants; Cardiovascular Diseases (Not Specified); Pulmonary Hypertension, Treatment of	II	Efficacy	Hypertension, pulmonary arterial
LIK-066 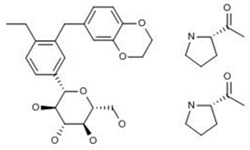	Novartis	SGLT-1 Inhibitors; SGLT-2 Inhibitors	Treatment of Female Sexual Dysfunction; Antiobesity Drugs; Metabolic Disorders (Not Specified); Heart Failure Therapy; Type 2 Diabetes, Agents for	II	Strategic (slow enrollment)	Heart failure
Sodium nitrite; AIR-001; S-2252; TV-1001; TV-1001-SR 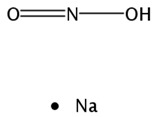	TheraVasc	Nitric oxide stimulant	Septic Shock, Hemorrhagic Stroke, Non-Opioid Analgesics, Neuropathic Pain, Poisoning, Acute Myocardial Infarction, Cerebrovascular Diseases, Ischemia, Peripheral Arterial Disease; Pulmonary Hypertension, Treatment of; Ulcers of the Extremities, Scleroderma Agents for; Cardioprotective Agents; Heart Failure Therapy; Antibacterial Drugs	II	Efficacy	Heart failure
TAK-536TCH #; Azilsartan/amlodipine/hydrochlorothiazide; TAK-536/amlodipine/hydrochlorothiazide	Takeda (Originator)	Signal Transduction Modulators; Insulin Sensitizers; Calcium Channel Blockers; Angiotensin AT2 Receptor Antagonists; Angiotensin AT1 Receptor Antagonists	Hypertension, Treatment of	III	Strategic	Hypertension, essential
Bococizumab *; L1L3; PF-04950615; RN-316	Pfizer (Originator)	Anti-PCSK9 (Proprotein Convertase Subtilisin/Kexin-Type 9)	Atherosclerosis Therapy; Disorders of the Coronary Arteries and Atherosclerosis; Lipoprotein Disorders, Treatment of	III	Efficacy	Hyperlipidemia, Hypercholesterolemia, familial
Losmapimod; 856553; GSK-AHAB; GW-856553; GW-856553X; SB-856553 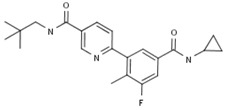	GlaxoSmithKline (Originator)	Mitogen-Activated Protein Kinase 14(MAPK14; MAPK p38 alpha) Inhibitors; Signal Transduction Modulators	Chronic Obstructive Pulmonary Diseases (COPD), Treatment of; Treatment of Renal Diseases; Neuropathic Pain, Treatment of; Atherosclerosis Therapy; Treatment of Disorders of the Coronary Arteries and Atherosclerosis; Antidepressants; Rheumatoid Arthritis; Lipoprotein Disorders, Treatment of; Antipsoriatics	III	Efficacy	Acute coronary syndrome

* Structures of the drugs are unavailable; # Fixed-dose combination of different drugs.
